# Research on digital copyright protection based on the hyperledger fabric blockchain network technology

**DOI:** 10.7717/peerj-cs.709

**Published:** 2021-09-17

**Authors:** Yanhui Liu, Jianbiao Zhang, Shupei Wu, Muhammad Salman Pathan

**Affiliations:** 1 Beijing Key Laboratory of Trusted Computing, Beijing, China; 2 Faculty of Information Technology, Beijing University of Technology, Beijing, China

**Keywords:** Digital copyright, Copyright protection, Blockchain, Consortium blockchain, Smart contract

## Abstract

With the recent development in network technology over a few years, digital works can be easily published online. One of the main issues in the field of digital technology is the infringement of digital works, which can seriously damage the data owners’ rights and affects the enthusiasm of the owners to create original work. Thus, more attention is required for the protection of digital copyright as it has a great impact on the development of society. Many digital copyright protection techniques were developed in the past, but still, there are many loopholes in the protection systems to be covered. The protection means are still relatively weak, timeliness is poor, infringement is frequent, a right determination is cumbersome, and the results are not ideal. Aiming at the mentioned problems, this paper proposes a protection technique, which can realize the automatic management of the complete digital rights life cycle on the blockchain using fabric's smart contract technology. The proposed system is based on blockchain technology, which leverages the distributed, tamper-proof and traceable characteristics of blockchain. The system uses smart contracts to manage the full life cycle of digital copyright. The test results show that the proposed system provides effective protection of the digital copyright system and can efficiently confirm the rights of digital copyright.

## Introduction

With the development of the internet, digital information technology is replacing books, newspapers, and offline exhibitions by playing the role of the main media in providing information in our daily life. However, there have some core issues in digital copyright systems:

1. The lack of transparency and a central database for organizing information about music, photos, and other copyrighted objects may cause serious problems when trying to identify the rights of owners to arrange the consequent use of these objects. The copyright owner’s information is scattered in the databases of publishers, record companies, collection associations, and other entities that have no incentive to share this information. Sometimes it is not available at all or its receipts are prohibitively expensive from a financial point of view. All these issues have brought huge transaction costs to users of these digital contents, and they sometimes even have to avoid using certain copyrighted works because of their unclear legal status. The lack of transparency and public information facilitates affects the authors and other rights owners, who do not accept compensation for using their works or sharing such compensation with intermediaries, such as a collective society, which retains a considerable portion of such compensation.
2. Right holders cannot effectively control the use of their work on the Internet. Digital copies of copyrighted works have the unique attributes of less copying and transmission costs close to zero. Digital copies are perfect copies, and each one is the seed for a further perfect copy. Natural obstacles to infringement no longer exist, such as the cost of copying and the decline in the quality of successive generations of copies in analog media. Nowadays, ordinary computer owners can easily copy, but a few years ago, such copying required a lot of investment and may even require crime. At the same time, there is no technical limit on the number of people who can access these digital works. No matter where the location is, as long as there is an Internet connection, they can access these works.
3. Considering a large amount of content on the Internet, its authors can be located in different jurisdictions, and as a result-subject of various legal procedures related to payment processing, it may be difficult to recover the license fee to use their works. In most cases, to facilitate the payment of license fees, the user and the right holder must sign a special agreement, which adds many transaction costs to both parties. Traditional creative sharing licenses are not suitable for the direct commercialization of works because they are royalty-free. Open source licenses for software distribution also have royalty-free distribution clauses. According to the first standard of open source, “licenses do not require royalties or other fees” to sell software. Therefore, software open source/creative sharing licenses are not customized for charging license fees. Their main goal is to promote the sharing of copyrighted works, subsequent legal use, sharing and re-use of related attributions, as well as exemption from potential responsibilities and guarantees. Achieving these goals is incompatible with the complicated procedures required to pay license fees in cash or through traditional financial institutions. Therefore, the price paid in the form of license fees still belongs to the category of exclusive licenses.

Traditional copyright protection methods cannot work in the field of digital publishing protection. Coupled with the general wrong consumption concept and the lack of awareness of copyright protection, digital products have become the “disaster area” of copyright protection (LIZ, 2020). Since 2018, the rise of knowledge payment has led more enterprises and individuals to participate actively in the creation and sharing of digital works (HTML5, application, soft text, games, print media, academic articles, etc.). However, with the rapid development of word recognition technology and pirated aggregation websites, the malpractice of serious personal information leakage, rampant piracy works, continuous copyright disputes, and the infringement of original works have emerged, which has greatly damaged the information security and vital interests of the original subject. The state has introduced some Digital Rights Management (DRM) technologies to protect the original works. Although they have a certain protective effect, still they are cracked and even turned into monopoly tools, which not only affects the creative work of the creators but also has a trend of piracy. The current situation of piracy infringement has become a serious concern in the field of digital works and the disputes caused by digital copyright problems cannot be underestimated.

At present, there are some problems in the digital industry, such as imperfect authorization mechanism, difficult copyright certification, long license issuing time, and high registration cost, which are not suitable for the current pursuit of low-cost network creation. The main problems are summarized in Table S1.

From the above description, it can be observed that there is an urgent need for advanced mechanisms to solve various problems existing in the registration, confirmation, and transfer of digital rights during the process of digital rights protection. One possible solution is the use of blockchain technology. The Blockchain technology originated in 2008 and was once used as the public transaction ledger of cryptocurrency which name is bitcoin. Researchers said that blockchain has the potential to be widely used in copyright protection and management. The digital copyright data is contained in the block. Each block is linked to the blockchain in the form of a timestamp through the hash pointer pointing to the hash value of the previous block. In this way, it guarantees that digital copyright information cannot be manipulated. Blockchain can significantly reduce the cost of digital copyright protection, improve efficiency, and provide new ways to collect evidence, trade digital assets, and protect the rights of copyright owners. This technology has greater transparency and centrality than the traditional publishing technology, and cannot be changed at will. Therefore, great changes have taken place in all aspects of copyright protection technology (Crosby et al., 2016). According to the distribution of blocks, blockchain technology can be divided into the public blockchain, alliance blockchain, and private blockchain. It has the characteristics of decentralization, openness, independence, security, and anonymity. It is widely used in the Internet of Things (IoT) and logistics, public service, digital copyright, insurance, and public welfare.

This paper proposes a digital copyright protection system based on the Hyperledger Fabric blockchain network. Through this system, users can perform digital copyright registration, transaction, inquiry, and cancellation operations without third-party interference, which effectively protects the security of copyright and maintains the stability of the transaction.

The rest of this paper is divided into the sections:

First, the paper provides an analysis of the existing problems of copyright protection. Secondly, the paper introduces the background of digital copyright protection including digital watermarking technology, blockchain technology, and Hyperledger fabric. After that, the paper discusses some related work in the area. Third, the proposed scheme section discusses the proposed technical framework for copyright protection based on blockchain technology, including the system architecture, data structure design, and system implementation. The paper also presents an experimental and evaluation section. Finally, the paper summarizes this research and points out the future research directions.

## Background

### Digital watermarking technology

Digital watermarking technology can be used in digital copyright protection (Sheppard, Safavi-Naini & Ogunbona, 2002; Deepa Merin Jose, Karuppathal & Vincent Antony Kumar, 2012; Woo, 2007). Digital watermarking technology is used to embed different iconic information as fingerprint information into digital media, and then distribute it to users. Digital fingerprint information can contain information such as the creator of digital media, the date of generation, and so on. Digital watermarking technology is generally having the characteristics of invisibility and robustness. The purpose of using watermarking technology is to prevent the copy and secondary distribution of illegal users by tracing them.

Although digital watermarking technology can solve many important problems in digital copyright protection, it still possesses some major problems:

1. The technology is cumbersome. There are many technical problems and loopholes. It is difficult to guarantee the unauthorized use of digital copyright works. Work registration, anti-counterfeiting recognition, customer authentication, and authorization, copyright management, and other technical methods of this technology are very complex.
2. Digital watermarking is not mature enough in technology. The extraction effect and the security of watermark information are not guaranteed.
3. Robustness is not strong enough. To ensure the robustness of digital watermarking technology, it is necessary to ensure that watermarking information must be difficult to erase. But in practice, any watermark can be removed, even for some complex watermark information that cannot be completely removed, part of the information can be removed.

### Blockchain technology

One of the most auspicious technologies in the new economy is distributed ledger technology, also known as “blockchain”. The World Economic Forum has estimated that more than 25 countries have invested in blockchain technology, applied for more than 2,500 patents, and invested 1.3 billion US dollars (Blockchain, 2020). Klaus Schwab, Founder and Executive Chairman of the World Economic Forum, gives the following definition of this technology: “The blockchain is a shared, programmable, cryptographically secure and therefore trusted ledger which no single user controls and which can be inspected by anyone (Shapiro & Varian, 1999).”

In addition to digital currency, blockchain technology has begun to expand in other areas in recent years, including digital copyright protection (Savelyev, 2017; Fujimura et al., 2015; Xu et al., 2017). Blockchain is a distributed account book with multi-node participation where the data cannot tamper. The scheme allows the node to link and record the transaction data through the Merkle tree for some time. Each block contains not only the transaction data, but also the timestamp identification and the parent block hash Blockchain. Blockchain development goes through the following three stages:

1. 1.0 era. Bitcoin is represented by digital currency applications. As a decentralized payment system, Bitcoin does not rely on any third-party organization, using cryptography technology and the whole network consensus to ensure the security of currency circulation.
2. 2.0 era. Ethereum represents the combination of digital money and intelligent contracts for decentralized applications. Besides serving as a circulation platform for digital money, smart contracts running on Ethernet Square can achieve more business (Christidis & Devetsikiotis, 2016).
3. 3.0 era. Hyperledger Fabric (Androulaki et al., 2018) is represented by permission control and authentication de-central application.

From the point of view of storage architecture, blockchain technology is a chain structure in which each block is connected in the order of timestamp, which involves techniques such as Hash function, Merkle tree, timestamp, and point to point (P2P) network (Wang et al., 2018; Di & Zhusong, 2018; Yang et al., 2018; Lee et al., 2008), etc.

**Figure 1:**
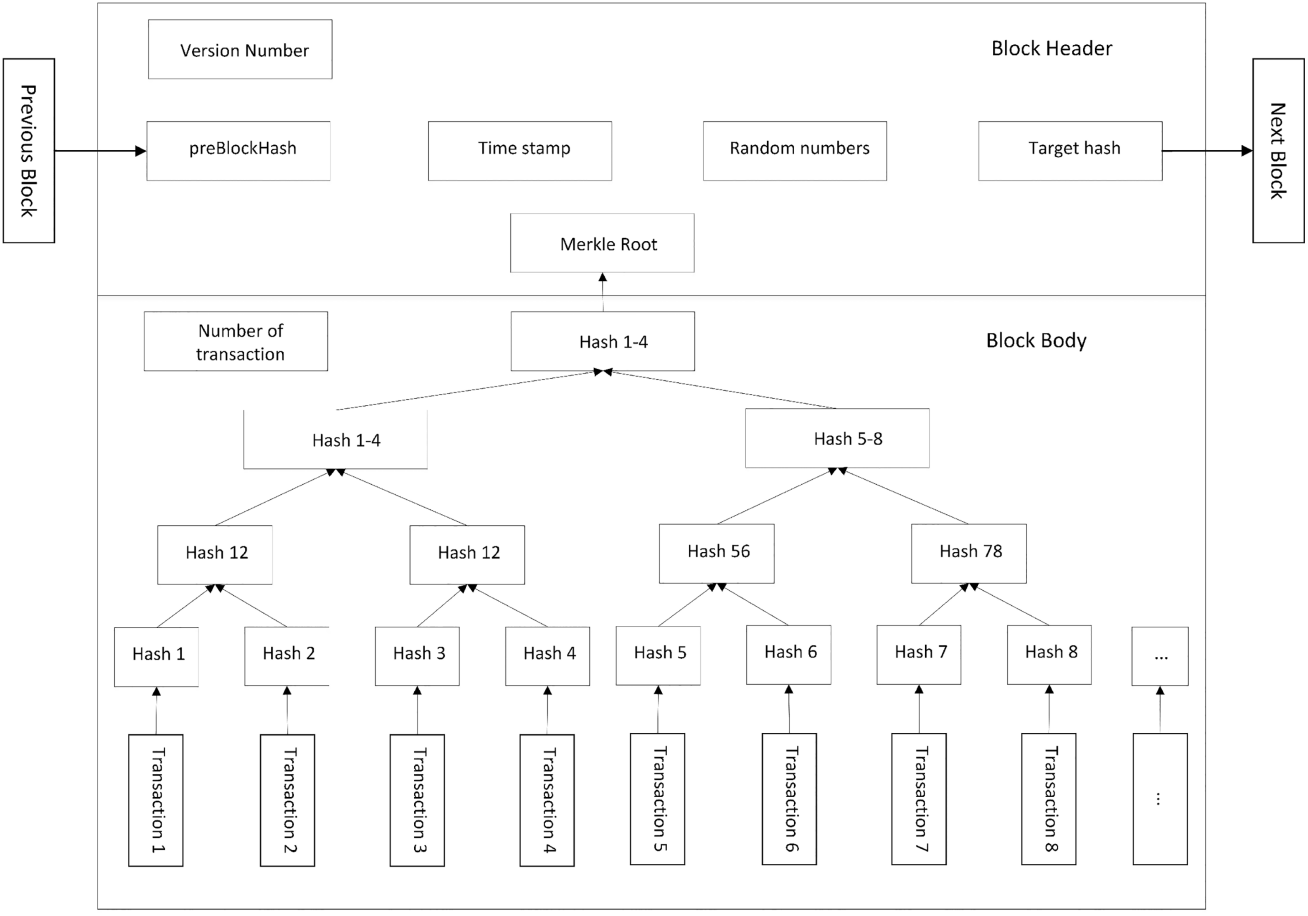
Block structure.

As shown in Fig. 1, the block structure can be divided into two parts: block header and block body. The block header contains the block version number representing the current block version, the hash value of the previous block header, the hash value of the Merkle tree root node generated from the transaction list, the timestamp generated by the block that can be accurate to seconds, the difficulty target representing the mining difficulty value, and the random value used in the mining process. The block body contains the number of transactions in the current block and hash values specific to each transaction, which are connected through a data structure called a Merkle tree.

The hash function also called a hash algorithm, is a method for creating small digital “fingerprints” for any kind of data. A hash function compresses a message or data into a summary that makes the amount of data smaller. This function creates a new hash value called (hash values, hash codes, hash sums, or hashes) fingerprints for the data. Mostly a hash value is a string of short random letters and numbers. Two hash codes rarely have hash conflicts in the input domain. It is very hard to temper or sniff the hash codes for particular data. Furthermore, if two hash values are different than the original input of the two hash values is also different. On the other hand, the input and output of a hash function are not uniquely related. Message-digest algorithm (MD5) (De Guzman, Sison & Medina, 2018), Secure Hash Algorithm 1 (SHA-1) (Ren et al., 2019) and RACE Integrity Primitives Evaluation Message Digest (RIPEMD) (Giechaskiel, Cremers & Rasmussen, 2018), are some common hash functions. The specific hash function used in the blockchain field is the Secure Hash Algorithm 256 (SHA256) function. A blockchain calculates a specific hash value by a hash function, and each block has a hash value of the previous block so that a chain-like data structure is formed between the blocks. The hash function has an important feature that when a hash value is generated for input and the input is modified even if it is only a very small part of it, the new hash value for the same input is completely changed.

According to whether the nodes of the blockchain need to be authenticated to participate in the recording of the block, the blockchain can be generally divided into three types, namely public chain, private chain, and alliance chain. The node of the public chain can be added to the blockchain system without any certification, and the private chain is only open to very limited internal individuals or entities. The nodes of the alliance chain are only open to members and limited third-party members of a particular group. Hyperledger Fabric is a very important alliance chain technology, and the paper use Hyperledger Fabric technology to implement copyright protection systems. The paper will introduce Hyperledger Fabric technology in the next section.

### Hyperledger fabric

In December 2015, the Linux Foundation announced the launch of the Hyperledger project, which aims to build an open platform to enable project members to work together, simplify business processes and promote the cross-industry application of blockchains. Unlike Bitcoin and Ethernet Square, which do not have any licensed public chains, the Hyperledger project only allows licensed members to join to have a certain trust base. Therefore, Hyperledger is not completely decentralized but can be considered as an alliance chain.

The fabric uses a modular architectural design. Its core features include the following six aspects:

1. Managing membership certificates using separate Fabric CA projects to facilitate the management of system members;
2. Classify nodes according to the functions of nodes in the system, such as endorsement nodes, consensus nodes, and submission nodes. It decouples the transaction-processing nodes functionally, and also decides the number of different nodes according to the needs of the business;
3. The consensus function is decoupled from other transaction processing links to improve scalability;
4. Setting up multiple channels to completely isolate the data in different channels;
5. Provide pluggable module design, consensus module, user rights management module, accounting mechanism, and so on. Developers can choose different modules according to the different needs of the business;
6. Provides intelligent contracts that support Go, Java, and Node.js languages, which are called system chain codes in the Fabric framework and can handle blockchain systems.

**Figure 2:**
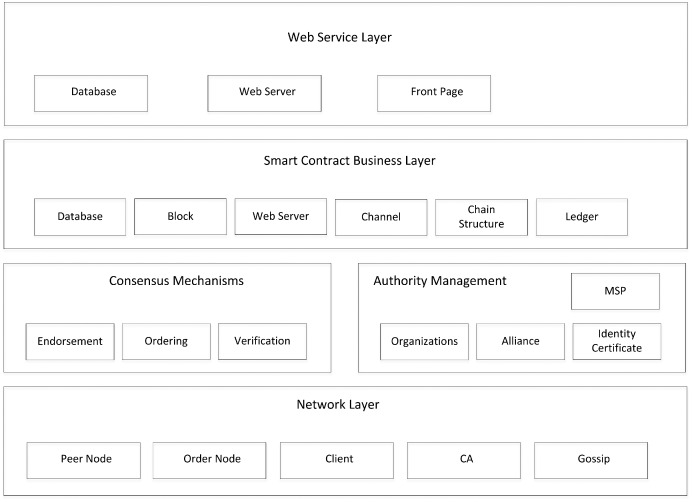
Structure of fabric.

The system architecture of Hyperledger Fabric (Klaokliang et al., 2018) is shown in Fig. 2, which can be divided into four layers from bottom to top:

1. Web application layer, facing the upper business application developer, mainly realizing the front-end module, directly facing the user through the front-end.
2. Smart contract business layer, for smart contract business developers, responsible for the implementation of chain code transactions and other related business code. As a core part of the blockchain, a smart contract is a computer protocol that is designed to spread, verify, or execute contracts in an information-based way. It allows trusted transactions without any third party. These transactions can be traceable and irreversible (Christidis & Devetsikiotis, 2016).
3. Consensus mechanism and authority management, for alliance and organization managers to achieve certificate management and consensus mechanism configuration.
4. The network layer, oriented to system administrators, implements P2P networks and provides the underlying capabilities for building blockchain networks, including nodes and services representing different roles.

Several basic concepts involved in the Hyperledger Fabric are described below:

1. Peer Node: the concept of node originated from P2P distributed network, represents a service or software that undertakes certain functions in the network. In the Hyperledger Fabric network, by functional role, the Peer node (Park, Hwang & Kim, 2018) can be divided into five types. The certificate node is responsible for issuing registration certificates to nodes and users; a submission node (submitter) is responsible for generating proposals and distributing them to the relevant endorsement node; endorsement node (the endorser) is responsible for the execution of the contract and endorsement response; a sort node (orderer) is responsible for sorting proposals, block packing; Confirm node (committer) is responsible for verifying the validity of the contract execution results, maintaining the blockchain and ledger structure. These roles are logically divided, not mutually exclusive. Usually, most nodes in the network have validation functions, while some nodes have an endorsement or sorting functions.
2. Chain code: the chain code in the Hyperledger Fabric (Linux Foundation, 2019), that is the intelligent contract mentioned above, is the medium for the upper application to interact with the underlying blockchain platform. Currently, Hyperledger Fabric support Go, Java, and other programming languages chain code. All chain codes inherit Init and Invoke interfaces. Init interface for initializing contracts, the interface is executed only once throughout the chain code life cycle. Invoke interface is essentially used to add and delete the underlying database of Blockchain. Different business logic can be distinguished according to the function name passed.
3. Channel: Channel (Gupta et al., 2018) provides a private channel for data exchange for nodes in the network. The node of the same channel can share or manage all books in the channel. One node can be added to multiple channels, managing multiple books, but the books of each channel are isolated. Therefore, the channel is a logical structure, which consists of physical nodes. Super books provide access channels and management channels.

## Related work

Many scholars have studied the use of blockchain technology to protect digital copyright (Wamba, Queiroz & Trinchera, 2020; Zou, Lv & Wang, 2019; Zhao & O’Mahony, 2018; Meng et al., 2018; Bellini, Iraqi & Damiani, 2020; Xiao et al., 2020; Zhao et al., 2020; Zhaofeng, Weihua & Hongmin, 2018; Peng et al., 2019a; Peng et al., 2019b; Liang et al., 2020; Chen, Zhang & Wei, 2020; Shi, Yi & Kuang, 2019; Miao et al., 2018; Ambili, Sindhu & Sethumadhavan, 2017; Ding et al., 2019; Tsai et al., 2017; Gürkaynak et al., 2018; Schönhals, Hepp & Gipp, 2018).

According to Wamba, Queiroz & Trinchera (2020), the use of blockchain technology can effectively reduce the market friction in the digital copyright trading market and improves the efficiency of licensing and the creative enthusiasm of creators. The intelligent contract in the blockchain has proved to be an effective way to confirm the right of copyright license and provide a higher level of transparency of capital flow for creators.

To address the problem of photo fraud, photo tracking, and copyright dispute, a photo forensics scheme based on blockchain is proposed by Zou, Lv & Wang (2019).

Zhao & O’Mahony (2018) have proposed an Ethernet-based application that protects music copyright and ensures copyright holders’ income rights.

A design scheme of copyright management system based on digital watermarking and its information is proposed by Meng et al. (2018). It combines digital watermarking, blockchain, perceptual hash function, fast response code, and InterPlanetary File System (IPFS).

Bellini, Iraqi & Damiani (2020) provides a comprehensive survey of the use of blockchains in distributed trust and reputation management systems (DTRMS) environments, including distributed picture sharing, picture copyright protection, and so on.

Xiao et al. (2020) provides the mathematical model of quadratic matrix transformation of an intellectual property transaction. A blockchain-based intellectual property protection algorithm is proposed.

A copyright trading protection method based on blockchain technology is proposed by Zhao et al. (2020). This method protects the complete copyright transaction process and identifies the attribute identification of the image content.

Zhaofeng, Weihua & Hongmin (2018) proposed a watermark and blockchain-based art image copyright management scheme, which uses image Arnold transformation to enhance security, image Discrete Cosine Transformation (DCT) if coefficient embedded watermark, enhance robustness.

Peng et al. (2019a) proposes a secure digital copyright management system based on Ethernet. A public chain-based system enables copyright owners and users to trade directly without resorting to central organizations.

A new method of cross-platform digital resource right recognition and infringement tracking based on intelligent contracts is designed by Peng et al. (2019b). At the same time, the intelligent contract is used to realize the right transaction.

Liang et al. (2020) proposed a knowledge copyright protection blockchain based on homomorphic encryption, which effectively solves the problems of low security of private data, low storage efficiency of transaction data, and low efficiency of cooperative supervision in the current knowledge copyright trading protection.

Chen, Zhang & Wei (2020) designed a decentralized video copyright protection protocol. The agreement provides copyright protection for videos on the blockchain, users can submit videos that may infringe copyright as a proposal, and the copyright committee can vote on the proposal to reach an agreement.

By using blockchain technology and Scale-invariant feature transform (SIFT) local feature extraction algorithm, Shi, Yi & Kuang (2019) implemented a new generation of image digital copyright systems. SIFT algorithm is used to extract the invariant features from images such as angles, brightness, and rotation, etc., and using IPFS for distributed storage of images’ copyright features. Finally, Hyperledger Fabric and smart contract are used to realize copyright registration, copyright transfer, and other functions. The author says it has the advantages of automatic similar infringement detection, decentralized storage, tamper-proof, and traceability.

New digital copyright works management system for protection, trading, and distribution based on Data Ownership Security Architecture (DOSA)was proposed by Miao et al. (2018) which can overcome the deficiencies of existing systems. DOSA is an architecture for data protection and application by using digital certification authentication and public key infrastructure.

DotBlockchain (Ambili, Sindhu & Sethumadhavan, 2017) was established by the Pledge Music Company, a start-up company in New York. The platform creates a new music format, called the .bc or dotBC. On the DotBlockchain platform, when an artist or rights holder publishes their musical work, they will create a .bc file instead of a standard audio file. Music data is bundled into a .bc file, including information on songwriters, performers, and the title of the music. Once this step is completed, all information is written into the blockchain and available to the public. Specialized players will use .bc rules to decode metadata and authorize or reject the play request.

Ding et al. (2019) combine digital copyright registration technology and blockchain technology to design a complete copyright registration protection application system. It focuses on the data storage protection of copyright and the security and reliability of blockchain technology. At the software level, the data is reliable and has a wide range of application prospects in the future.

Tsai et al. (2017) proposed a blockchain-based model and framework for microfilms’ intellectual property (IP) protection in China, especially for microfilms’ scripts and names. Both the name and the script can be used to identify other microfilms, and they can be stored in the blockchain and database. Lightweight binary watermarks are used in this model, to protect the microfilm script (such as story outlines, outlines, scenes described by scenes, etc.).

Gürkaynak et al. (2018) proposed a blockchain-based solution to facilitate the operation of the IP offices, strengthen the custom procedures to detect counterfeit products, and improve the efficiency of intellectual property management for right holders. They also put forward some suggestions to pave the way for the development of blockchain technology, increase the number of people using the technology, and successfully integrate it into various services and registration/transaction channels in IP management.

Schönhals, Hepp & Gipp (2018) proposes a blockchain-based approach that can protect developed ideas and early concepts in product design and development. To guarantee both proof-of-existence and proof-of-origin, the origin stamp decentralized trusted timestamp service generates a specific hash from each digital artifact stored and embedded in the Bitcoin blockchain. Once this unique fingerprint is embedded in the transaction in the basic blockchain network, it can be proven where the specific contribution comes from due to the characteristics of the blockchain architecture.

However, these studies are focusing on a single aspect, without using a smart contract to manage the whole life cycle of copyright automatically. However, these achievements are also beneficial to the protection of digital rights (Borrego et al., 2019; Sagar, Jhaveri & Borrego, 2020; Mahatpure, Motwani & Shukla, 2019; Chaturvedi & Shukla, 2020; Jiang et al., 2020).

## Proposed scheme

### System architecture

**Figure 3:**
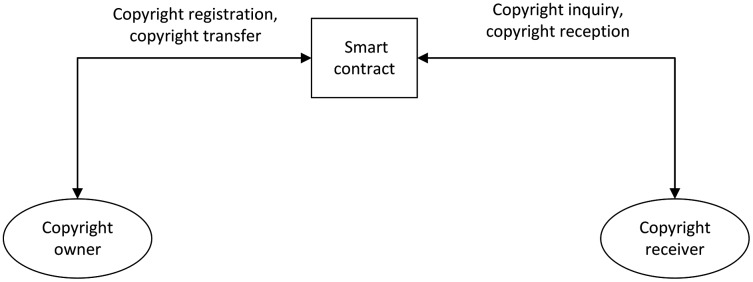
System architecture.

As discussed earlier, the paper proposes the following system structure, as shown in Fig. 3.

The system includes the copyright owner, the copyright receiver, and the distributed deployment of smart contracts, which can achieve a variety of business functions.

**Figure 4:**
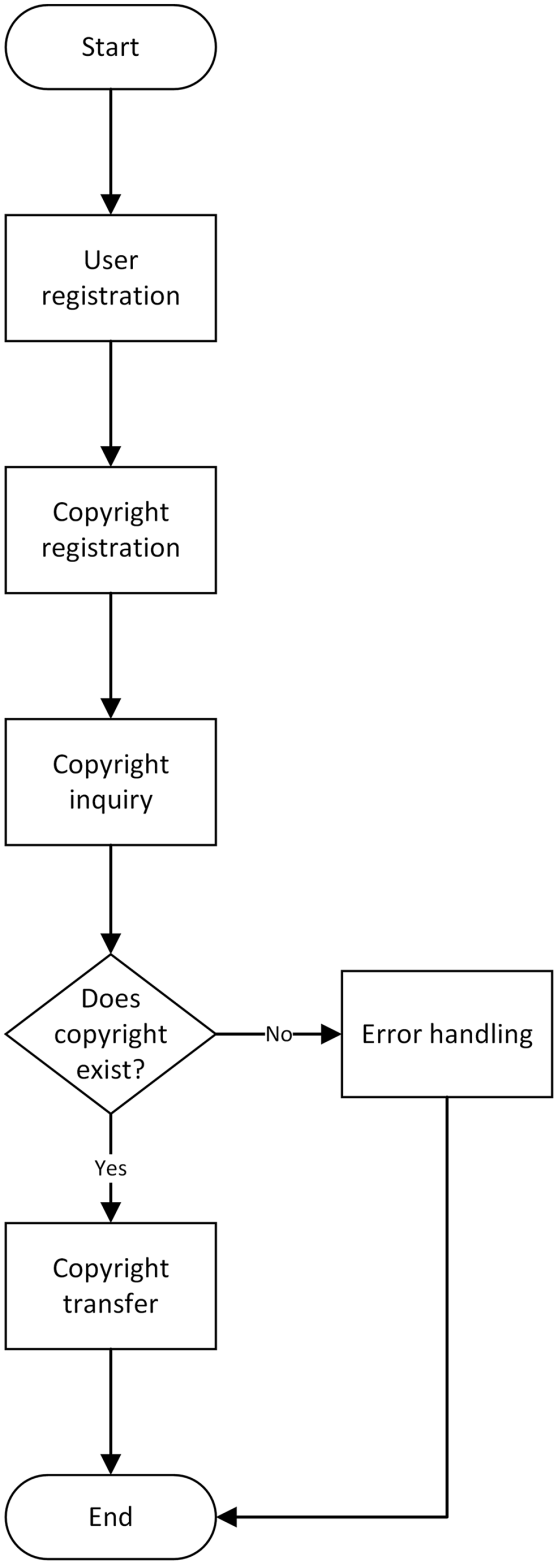
System workflow.

The workflow of the whole system is shown in Fig. 4.

The detailed description of the whole process is as follows:

1. User registration: all users must register before using the system;
2. Copyright registration: the copyright owner registers the copyright of the digital works on the system. During the registration process, the fingerprint information of the digital works needs to be extracted and stored on the blockchain;
3. Copyright query: before purchasing the copyright, the copyright receiver needs to call the smart contract to verify and query whether the copyright to be purchased is owned by the copyright owner *i.e*., to confirm the right of digital copyright. If the right is not confirmed, the process will be terminated, otherwise, it will go to the next step;
4. Copyright transfer: after the copyright receiver purchases the copyright and pays the corresponding consideration, the copyright owner calls the smart contract to transfer the copyright to the copyright receiver, and the whole process ends.

**Figure 5:**
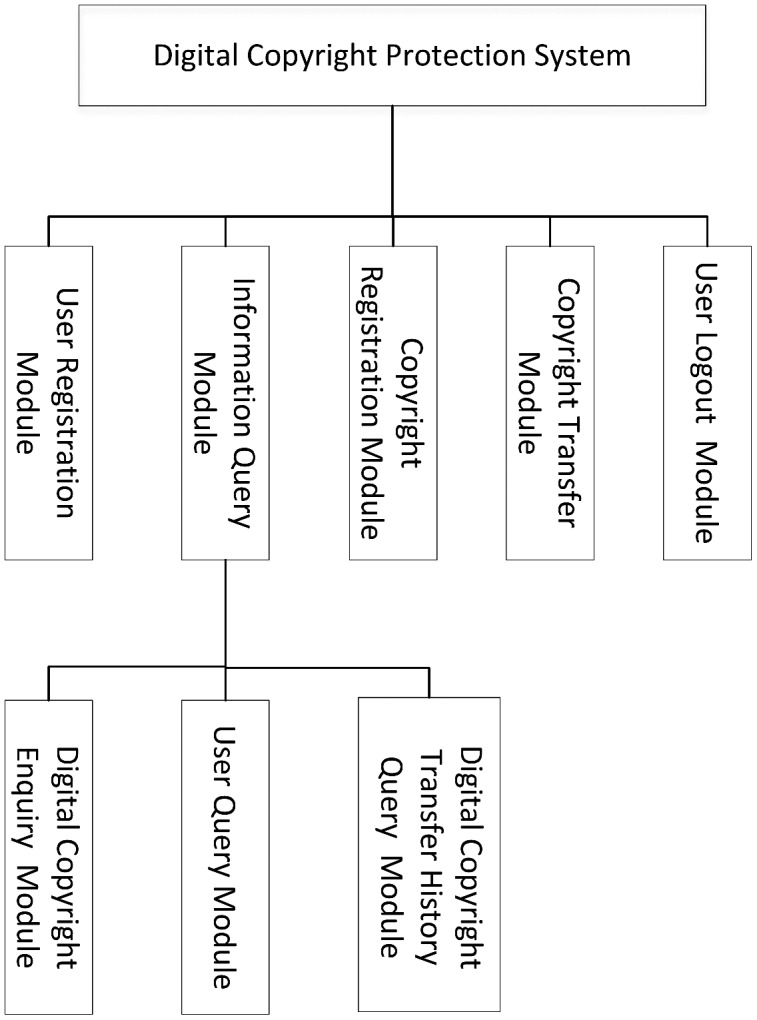
Module structure.

According to the specific requirements of digital copyright protection, this research has implemented the following five modules as shown in Fig. 5: user registration module, digital copyright registration module, information query module (including digital copyright query module, user query module, and digital copyright transaction history query module), digital copyright transfer module and user cancellation module.

#### User management

User management is mainly divided into the following two modules:

(1) User registration module

**Figure 6:**
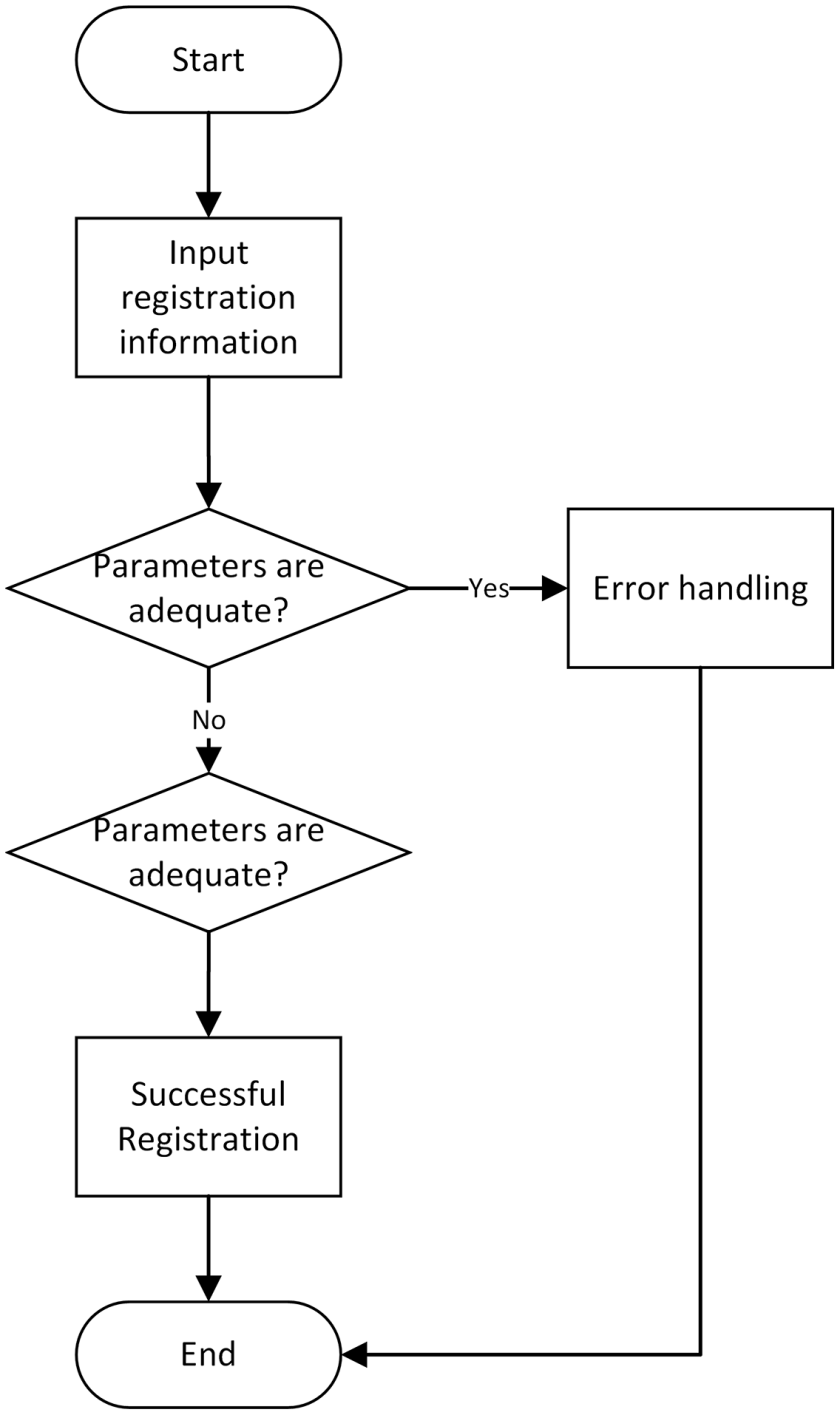
User registration flow chart.

The user registration module requires the user to fill in his name and Identity document (ID). The ID attribute cannot be repeated for the registered user. The user can also selectively enter basic information such as the mobile phone number and the work unit. The flow chart of the user registration module is shown in Fig. 6.

(2) User logout module

**Figure 7:**
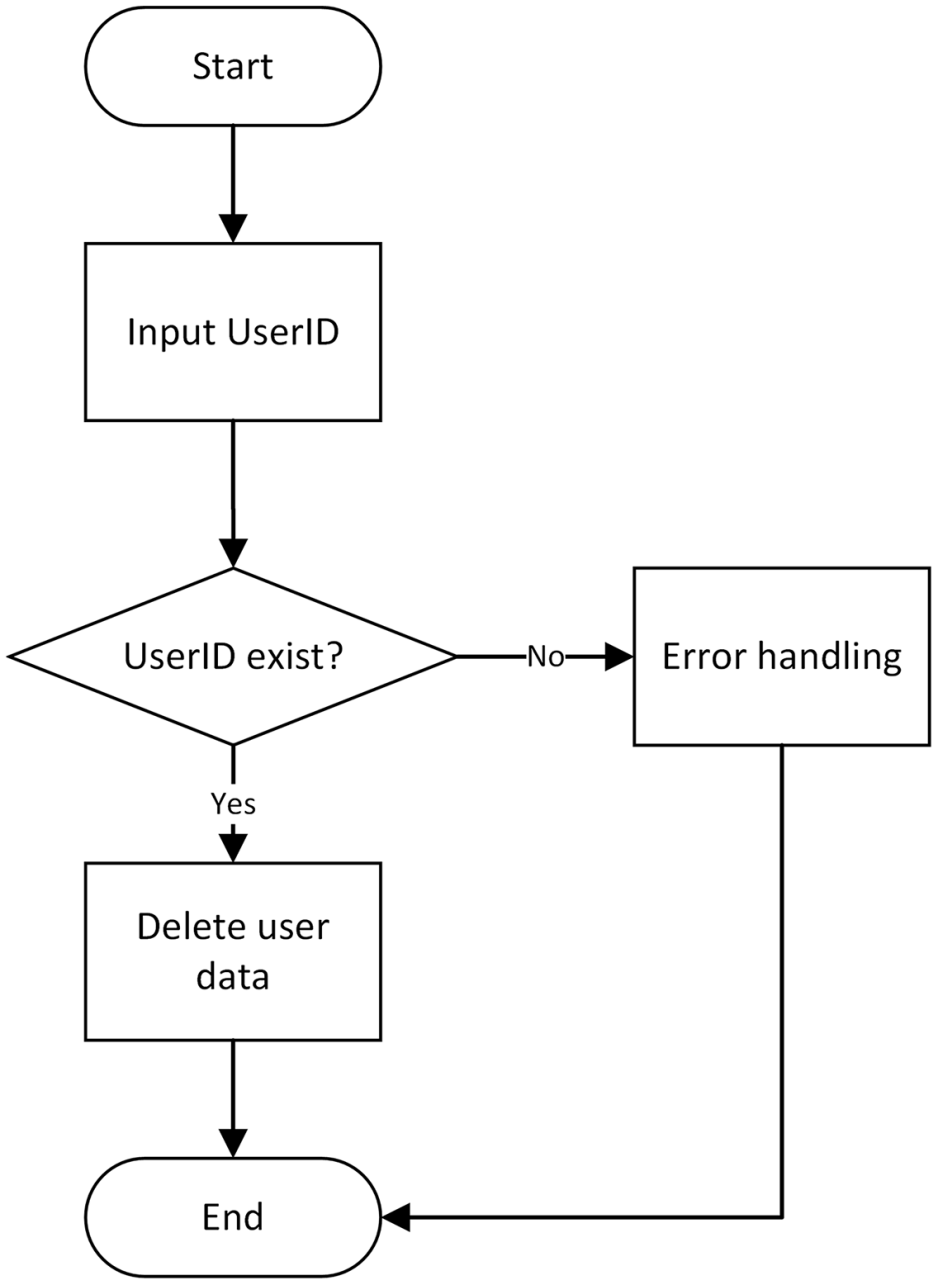
Copyright registration flow chart.

If the users need to cancel the accounts, they only have to enter the ID of the required cancellation account to cancel it. After account cancellation, the digital copyright also gets canceled. The flow chart of user logout is shown in Fig. 7.

#### Copyright management

Copyright management is mainly divided into the following three modules:

(1) Information query module

**Figure 8:**
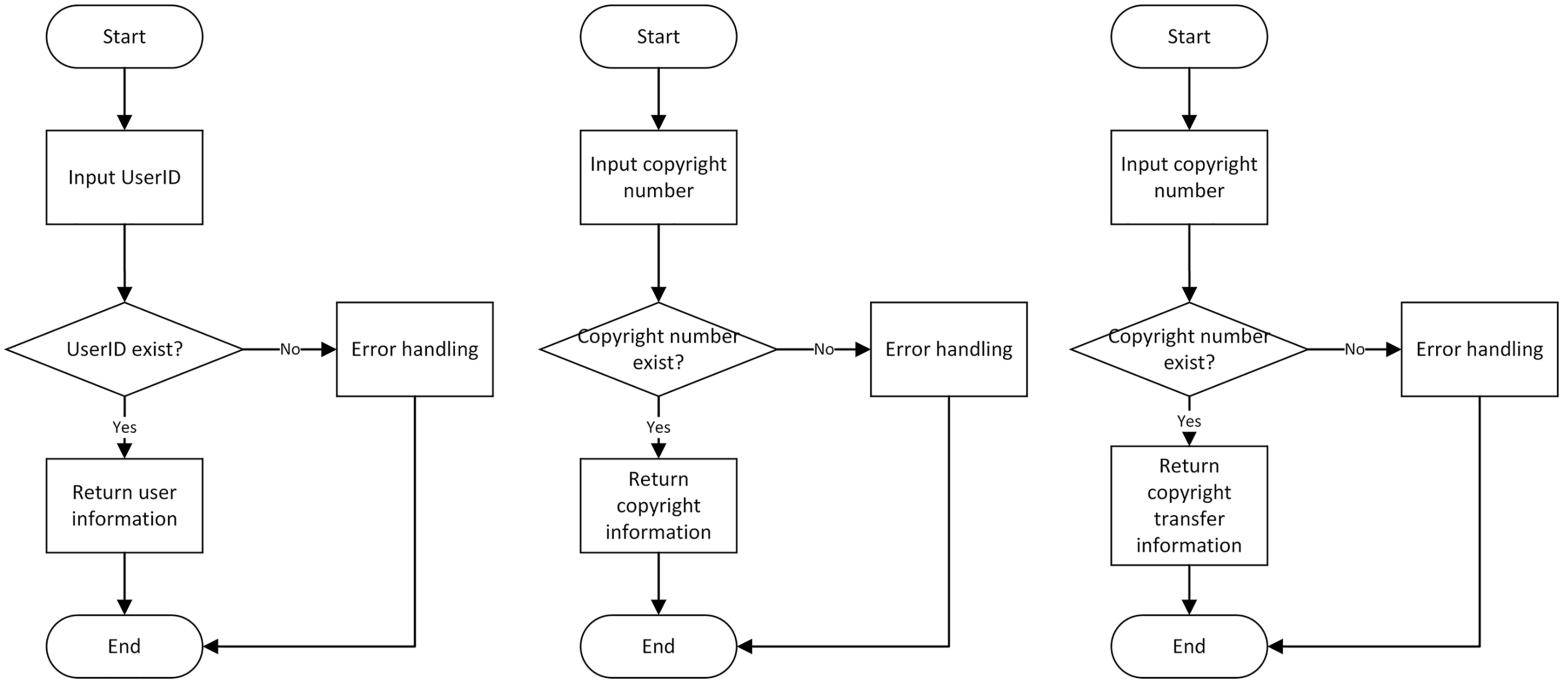
Query flow chart. The diagram describes the process diagram related to the query, including finding the information related to the user, finding the information related to the copyright and finding the information related to the copyright transfer.

Users can choose three query modes to inquire information: digital copyright information query, user query, and digital copyright transfer record query. If the user enters the copyright number for the information query, it can query the relevant information of the copyright and the current owner of the copyright. When the user enters the user ID for the information query, he can query the user’s relevant information and all the copyrights currently owned. When the user makes a copyright transfer history query, he can view all transfer records from registration to date. The flow chart of the query module is shown in Fig. 8.

(2) Copyright registration module

**Figure 9:**
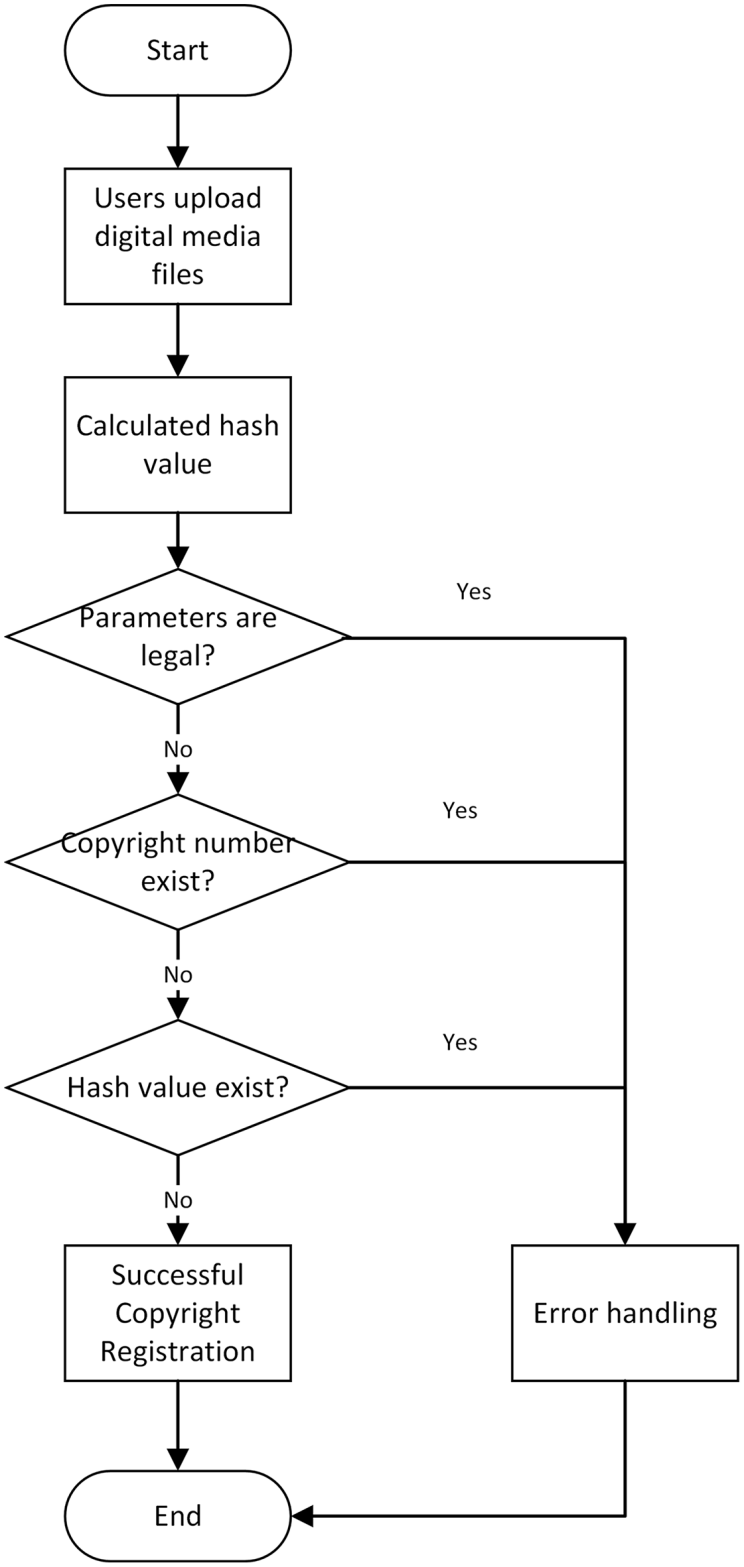
Copyright registration flow chart.

Users need to enter the digital copyright number (the copyright number cannot be the same value as the registered copyright), the copyright name, and the copyright type, and upload the corresponding digital file. Another program converts the uploaded digital media files, and the transferred hash values are stored on the blockchain with copyright information. The flow chart of the copyright registration module is shown in Fig. 9.

(3) Copyright transfer module

**Figure 10:**
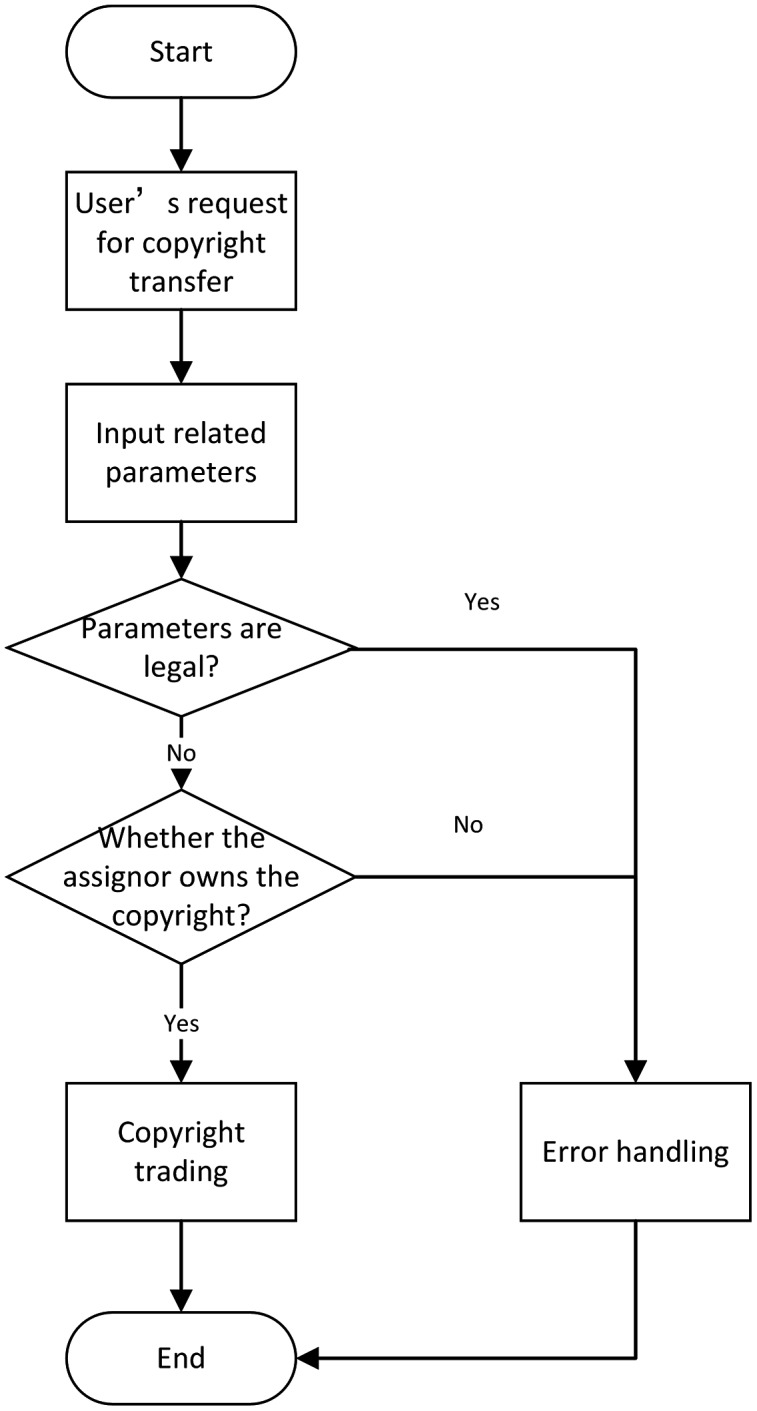
Flowchart of copyright transfer.

The user is required to enter the copyright number, ID of the copyright owner, and the copyright granter ID for the copyright to be transferred. During this step, the copyright transfer will also get updated at the same time. Furthermore, the transferred copyright is removed from the transferor side and is added to the copyright information owned by the grantor. The flow chart of the copyright transfer module is shown in Fig. 10.

### Data structure design

**Figure 11:**
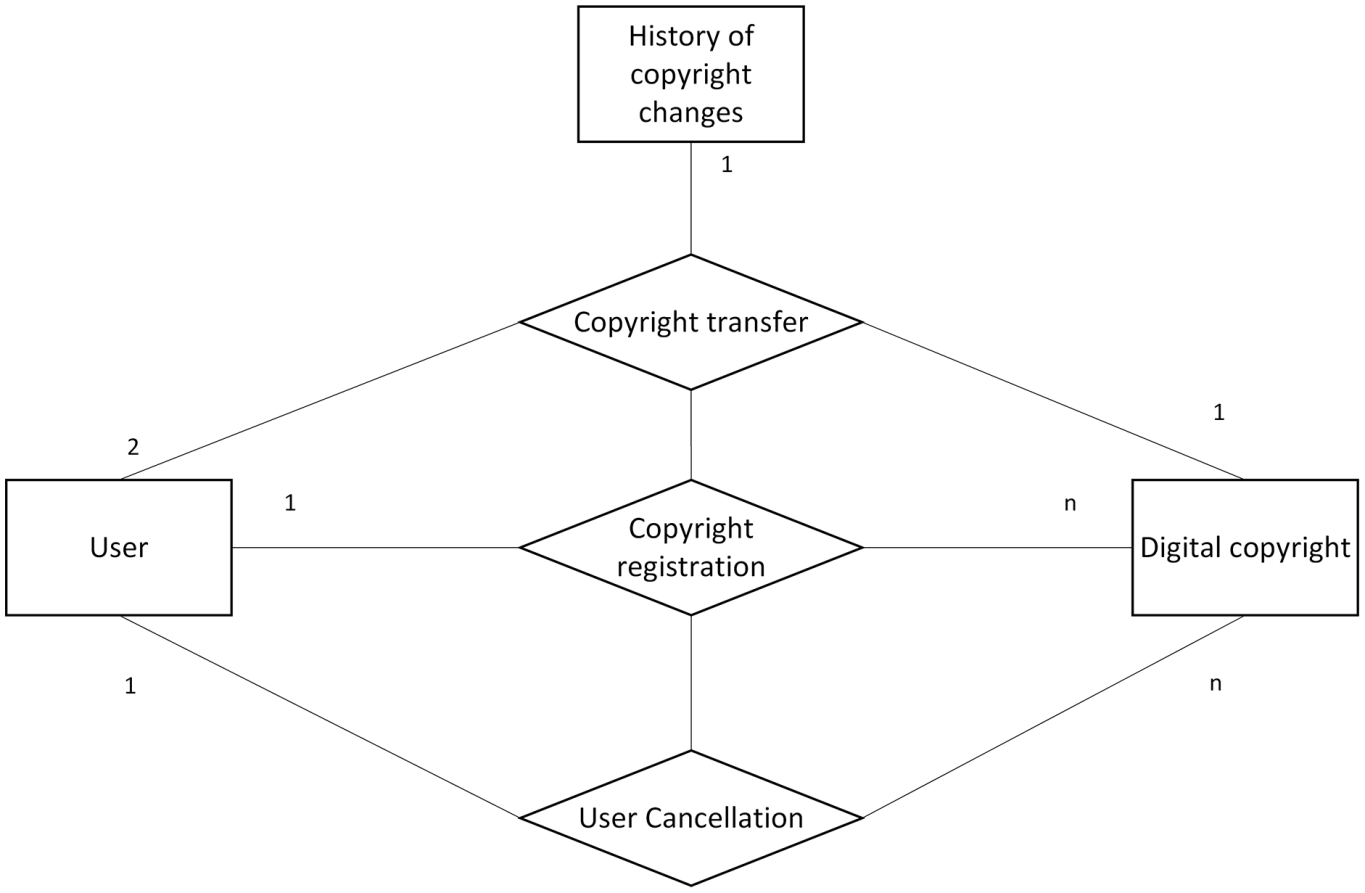
Entity relationship diagram.

For the storage system of digital copyright protection, the required parameters of each module are designed according to the traceability requirements of each module. The system involves three entities: user, digital copyright, and copyright change history. The relationship is shown in Fig. 11.

All entity-specific parameters in the system are designed as shown in Tables S2–S4.

The user can query the user’s information by entering the user ID, user’s basic information, and the copyright number of the copyright owned by the user, which is the interactive object of this system. In the copyright transfer operation, the user also needs to enter the original owner of the copyright and the user ID of the grantor.

Digital copyright is the core entity in this system and almost all the user’s operations involve this entity. The Metadata parameter of digital copyright is the hash value of digital media files uploaded by users after digital fingerprint extraction. This prevents the files uploaded by users from being tampered with.

When the user performs a copyright transfer operation, the corresponding copyright change records get generated with the copyright ID, and then all the transfer records of particular copyright can be viewed in the subsequent copyright transfer query.

Each ledger maintained by peer nodes in the Blockchain system consists of two parts: World state and Blockchain. The world state stores the latest values of all the transaction states, and the Blockchain system stores the data in the form of blocks.

### System implementation

The Hyperledger Fabric network uses a single-machine multi-node network configuration with the start-up type solo. The network configuration of the system is networked with a sorting service node and peer nodes under two organizations, where each organization includes two peer nodes. In this implementation of the system, the listening port number of the orderer sorting service is set to 7050.

In the Hyperledger Fabric, the smart contracts are called chain codes. The smart contract will be installed and instantiated on a peer node service by an authorized member. Then, some business personnel can use a client that can execute the Fabric-Software Development Kit (Fabric-SDK) to interact with the peer node service to obtain the smart contract call.

The following is the structure defined in the smart contract, and the specific parameters are as follows:

  *type Digitalright struct {*

      *Name string `json: “name”`*

      *Id string `json: “id”`*

      *Type string `json: “type”`*

      *Metadata string `json: “metadata”`*

    *}*

  *type DigitalrightHistory struct {*

      *DigitalrightId string `json: “digitalright_id”`*

    *OriginOwnerId string `json: “origin_owner_id”`*

      *CurrentOwnerId string `json: “current_owner_id”`*

    *}*

  *type User struct {*

    *Name string `json: “name”`*

    *Id string `json: “id”`*

  *Tel string `json: “tel”`*

      *Gender string `json: “gender”`*

    *Address string `json: “address”`*

      *Password string `json: “password”`*

  *Digitalrights []string `json: “digitalrights”`*

  *}*

In this work, chain-code is used to implement complex business logic. In the proposed methodology, the Go programming language is used to develop the chain code. All functional interfaces that are included in the smart contract are shown in Table S4.

The Smart contracts will run on public channels and all the users can write their identity and copyright information into the blockchain to complete the copyright transactions. After the data is written into the blockchain network, it cannot tamper and it is added to the public channel. All peer nodes of the channel can also query all transaction information in the channel. The following steps briefly introduce the five main algorithms involved in the smart contract:

1. Algorithm 1 User registration

*Input: username, userId, tel, address, password, gender*

*Output: if success, return transaction data else throw an exception*

Description: Before writing the user information to the blockchain, the user information is authenticated. If all the steps are passed normally, the user Register function will call the JSON.Marshal interface for the data sequence and then stub.PutState will be called to store the data on the blockchain.

2. Algorithm 2 Registration of copyright information

*Input: digitalrightName, digitalrightId, digitalrightType, metadata, ownerId*

*Output: if success, return transaction data else throw exception*

Description: Before writing the copyright information into the blockchain, it will first verify the legality of the copyright information and checks whether the copyright (digitalrightId) number already exists or not. If the copyright information is legal and valid, it will verify whether the copyright owner (ownerId) has been registered already. If so, the registered user will first serialize and then deserialize the user’s copyright information to update user information. After completing the update process, a record will be created and added to the copyright transaction history.

3. Algorithm 3 Copyright transfer

*Input: digitalrightId, ownerId, currentownerId*

*Output: if success, return transaction data else throw an exception*

Description: While performing the copyright transfer, it will first check whether the copyright number (digitalrightId) of the copyright to be transferred exists. If it exists, it will match whether the current owner is currently entered (ownerId) and whether the transaction user (currentownerId) is a registered user. After completing the verification, the acquired user information gets serialized and then deserialized to update the copyright information. Finally, a copyright change record of the transaction will be inserted.

4. Algorithm 4 Information query

*Input: digitalrightId, ownerId, queryType*

*Output: if success, return query result else throw an exception*

Description: The query function is divided into three parts: user query, copyright query, and copyright transaction record query. When the user performs a user query or copyright query, the query ID (ownerId) or copyright number (digitalrightId) entered by the user is verified. Registered users or registered copyrights will get the corresponding query information. During the copyright transaction record query, the operations will be performed according to the type of query requested by the user (queryType).

5. Algorithm 5 User Delete

*Input: userId*

*Output: if success, return transaction data else throw an exception*

Description: The user logout module will first verify the entered user ID (userId) to get it logged out from the system. The stub.DelState interface is called to delete user information and at the same time, it will reverse the sequence of the user information and then again the stub.DelState is called. Finally, the interface deletes the copyright owned by the user.

## Experiments and evaluation

In this work, the authors have proposed to design and implement a digital copyright protection system based on the Hyperledger Fabric blockchain network. Through this system, users can perform digital copyright registration, transaction, inquiry, and cancellation operations without third-party interference, which effectively protects the security of copyright and maintains the stability of the transaction.

### Experimental environment

This paper used the following testing environment in Tables S5 and S6:

The system was built using Hyperledger Fabric version 1.0. The paper used the “docker-compose -f docker-orderer.yaml up –d” and “docker-compose -f docker-peer.yaml up –d” to start the ordering service node and peer node. “docker ps” command was used to check whether the container is successfully started. The container query result is shown in Fig. S1, where the ordering service and peer node both started successfully.

After all the containers were started successfully, the next step was to create a channel (channel) and join the operation. Since there is no third-party SDK to make the join, the joining of the channel only needs to enter the started client (CLI) to operate. The channel was created using the command “Peer channel create -o orderer.example.com:7050 -c mychannel -t 50 –f ./channel-artifacts/mychannel.tx”. This command defines the channel ID to be created as mychannel. After the channel is created, the file is blocked after joining, and the start of the fabric network is completed.

### Test case

When designing the test data, all functions of the system should be tested. At the same time, possible events should be taken into consideration so that the system can be fully tested, to judge whether the system meets the design requirements. Tables S7–S9 shows system construction data (the construction data is only for testing the functions implemented by the smart contract).

### Test results

During the user registration function test, the “peer chaincode invoke -C mychannel -n mychannel -c‘{“Args”: [“userRegister”, “Piff”, “522003”]}”’ format commands are used for network interaction. When the user's input parameters meet the requirements, the system will prompt success, else the system will report the corresponding error. The test results are shown in Figs. S2–S4.

When testing the copyright registration function, use peer chain code invoke -C mychannel -n mychannel -c‘{“Args”: [“assetEnroll”, “Manta”, “20191101”, “213214123123”, “522001”]}’ format The command interacts with the system. Similarly, when the user inputs parameters that meet the requirements, the system will prompt success. When the user enters an existing copyright number, a non-existent user ID, or does not enter enough parameters, the system will report a corresponding error. The test results are shown in Figs. S5–S8.

During the test of copyright transfer functions, the command of peer chain code invokes -C mychannel -n mychannel –c‘{“Args”: [“assetExchange”, “user1”, “522001”, “522002”]}’ are used where the user Sean The “Blooming” owned is transferred to the user Lexie. After the operation is successful, the target copyright and the information of both users get updated. When the user enters a non-existent copyright number, a non-existent user ID or it does not enter enough parameters, the system will report the error. The test results are shown in Figs. S9–S12.

The query function involves three parts: user, copyright, and copyright transaction record query. The logic of the query function is similar, that’s why only the results of successful queries are displayed. As shown in Fig. S13, the query for the user with ID “522002” has returned the user’s basic information and all the copyright number information. Figure S14 shows the result of querying the copyright number “19980722”, which correctly returns the copyright name, ID, and hash value of it. Figure S15 shows the results of querying the copyright number for which the results are returned including all transaction records of “19980722” (including copyright registration).

### Result analysis

We have presented a comparative analysis in which we have compared our proposed methods with already developed similar methods to prove the efficiency. Table S10 shows a comparison of the proposed model with other similar models. The comparative analysis is performed based on five aspects.

1. Whether it supports the whole life cycle management of copyright;
2. Whether a lot of calculation is needed;
3. Whether it supports the digital copyright protection of different types of files;
4. Whether the data storage on the link needs to pay a fee;
5. Whether it supports smart contracts or not.

It can be seen from the above tables that, only the proposed scheme supports the whole life cycle management. Other schemes need a lot of calculation for using public blockchain (Zhao & O’Mahony, 2018) or using homomorphic encryption (Liang et al., 2020) or using watermarks technology (Ambili, Sindhu & Sethumadhavan, 2017). Only this scheme support protection of different types of files. The scheme is needed to pay the fee by using Ethereum (Zhao & O’Mahony, 2018). The other compared schemes do not support smart contracts (Liang et al., 2020; Ambili, Sindhu & Sethumadhavan, 2017; Tsai et al., 2017).

According to the previous comparative analysis, the system can realize the automatic management of the whole life cycle of digital rights on the blockchain by using fabric’s smart contract technology. The whole life cycle of digital rights includes the registration, transfer, and query of digital rights and so on.

Because the data on the blockchain has the function of distributed storage and tamper proof, all the data about the whole life cycle of digital rights are stored on the blockchain. In this way, the data stored in the system has authority and credibility, preventing the theft of digital rights, and realizing the effective protection of digital rights.

## Conclusion and future work

With the rapid development of digital publishing, digital copyright infringement is becoming more and more serious. The frequent occurrence of infringement cases has severely dampened the enthusiasm of original creators. Due to the tamper-proof, decentralized, and other features of blockchain, this paper uses these features to build a copyright protection system.

Firstly, this paper analyses the problems existing in digital copyright protection. Secondly, this paper introduces digital watermarking technology, blockchain technology, and the work of other researchers. Thirdly, a digital rights protection system based on an alliance chain structure is proposed. The system uses smart contracts to automatically manage the entire life cycle of digital rights without any trusted third party. Then the prototype design and experiment of the system are carried out, and the experimental results are compared with other schemes from five different aspects. The five aspects include if support whole life cycle management copyright, need pay fee, support smart contract and so on. The comparison results show the superiority of the scheme, and finally, the conclusion is given.

In the future, this study will reshape the business process of digital rights protection, implement some important steps on mobile terminals, and comprehensively consider security and convenience, and also find the infringement of copyright by using big data technology is an interesting work in the future.

## Supplemental Information

10.7717/peerj-cs.709/supp-1 Supplemental Information 1 Related configuration files for building blockchain networks. There are related certificates, configuration files, block chain network initialization, startup, end script files and so on.

10.7717/peerj-cs.709/supp-2 Supplemental Information 2 Chaincode source file. Implementation code for copyright protection based on smart contracts

10.7717/peerj-cs.709/supp-3 Supplemental Information 3 Disadvantages of existing methods.

10.7717/peerj-cs.709/supp-4 Supplemental Information 4 User entity parameters.

10.7717/peerj-cs.709/supp-5 Supplemental Information 5 Digital rights entity parameters.

10.7717/peerj-cs.709/supp-6 Supplemental Information 6 Digital copyrights trade history entity parameters.

10.7717/peerj-cs.709/supp-7 Supplemental Information 7 Table of simulation hardware environment.

10.7717/peerj-cs.709/supp-8 Supplemental Information 8 Simulation software environment table.

10.7717/peerj-cs.709/supp-9 Supplemental Information 9 User test samples.

10.7717/peerj-cs.709/supp-10 Supplemental Information 10 Digital copyrights test samples.

10.7717/peerj-cs.709/supp-11 Supplemental Information 11 Digital copyrights transaction records test sample.

10.7717/peerj-cs.709/supp-12 Supplemental Information 12 Comparative analysis of safety attributes.

10.7717/peerj-cs.709/supp-13 Supplemental Information 13 Docker query result.

10.7717/peerj-cs.709/supp-14 Supplemental Information 14 User successfully registered.

10.7717/peerj-cs.709/supp-15 Supplemental Information 15 Enter duplicate ID.

10.7717/peerj-cs.709/supp-16 Supplemental Information 16 Not enough parameters entered.

10.7717/peerj-cs.709/supp-17 Supplemental Information 17 Successful registration of copyright.

10.7717/peerj-cs.709/supp-18 Supplemental Information 18 Enter the existing copyright number.

10.7717/peerj-cs.709/supp-19 Supplemental Information 19 Enter a user ID that does not exist.

10.7717/peerj-cs.709/supp-20 Supplemental Information 20 Not enough parameters entered.

10.7717/peerj-cs.709/supp-21 Supplemental Information 21 Copyright successfully transferred.

10.7717/peerj-cs.709/supp-22 Supplemental Information 22 The transferor does not own the target copyright.

10.7717/peerj-cs.709/supp-23 Supplemental Information 23 Participants did not register for the test.

10.7717/peerj-cs.709/supp-24 Supplemental Information 24 Not enough parameters entered.

10.7717/peerj-cs.709/supp-25 Supplemental Information 25 User query test.

10.7717/peerj-cs.709/supp-26 Supplemental Information 26 Copyright query test.

10.7717/peerj-cs.709/supp-27 Supplemental Information 27 Copyright transaction records query test.

## Additional Information and Declarations

### Data Availability

The following information was supplied regarding data availability: The raw measurements are available in the Supplementary Files.
