# Supplementary material for: Research on digital copyright protection based on the hyperledger fabric blockchain network technology
Source: PeerJ Comput Sci. 2021 Sep 17;7:e709. doi: 10.7717/peerj-cs.709 (PMC8459789; doi:10.7717/peerj-cs.709)
Supplement: Supplemental Information 3 [file peerj-cs-07-709-s003.docx]

| Name | Disadvantages |
| --- | --- |
| Digital watermarking technology | Easy to crack |
| Digital copyright management platform | Protection cycle is long |
| Data storage based on third-party | Vulnerable to attack and tampering |
