# Supplementary material for: Research on digital copyright protection based on the hyperledger fabric blockchain network technology
Source: PeerJ Comput Sci. 2021 Sep 17;7:e709. doi: 10.7717/peerj-cs.709 (PMC8459789; doi:10.7717/peerj-cs.709)
Supplement: Supplemental Information 4 [file peerj-cs-07-709-s004.docx]

| Variable name | Type of variable | Is it necessary | Description |
| --- | --- | --- | --- |
| Name | String | Yes | User Name |
| ID | String | Yes | User ID |
| Tel | String | No | Telephone |
| Digitalrights | [] String | No | Digital copyrights Information |
| Gender | String | Yes | Gender |
| Address | String | No | User Address |
| Password | String | Yes | User Password |
