# Supplementary material for: Research on digital copyright protection based on the hyperledger fabric blockchain network technology
Source: PeerJ Comput Sci. 2021 Sep 17;7:e709. doi: 10.7717/peerj-cs.709 (PMC8459789; doi:10.7717/peerj-cs.709)
Supplement: Supplemental Information 5 [file peerj-cs-07-709-s005.docx]

| Variable name | Type of variable | Is it necessary | Description |
| --- | --- | --- | --- |
| Name | String | Yes | Digital copyrights name |
| ID | String | Yes | Digital copyrights ID |
| Type | String | Yes | Media file type |
| Metadata | String | Yes | Media file hash value |
| Time | String | Yes | Media file upload time |
