# Supplementary material for: Research on digital copyright protection based on the hyperledger fabric blockchain network technology
Source: PeerJ Comput Sci. 2021 Sep 17;7:e709. doi: 10.7717/peerj-cs.709 (PMC8459789; doi:10.7717/peerj-cs.709)
Supplement: Supplemental Information 6 [file peerj-cs-07-709-s006.docx]

| Variable name | Type of variable | Is it necessary | Description |
| --- | --- | --- | --- |
| DigitalrightId | String | Yes | Digital copyrights ID |
| OriginOwenerId | String | Yes | Origin Owner ID |
| CurrentOwenerId | String | Yes | Current Owner ID |
| Time | String | Yes | Trading time |
