# Supplementary material for: Research on digital copyright protection based on the hyperledger fabric blockchain network technology
Source: PeerJ Comput Sci. 2021 Sep 17;7:e709. doi: 10.7717/peerj-cs.709 (PMC8459789; doi:10.7717/peerj-cs.709)
Supplement: Supplemental Information 7 [file peerj-cs-07-709-s007.docx]

| CPU | Memory size | Size of hard disk storage space |
| --- | --- | --- |
| Inter(R) Core(TM) i7 8750H CPU @2.20GHz | 8GB | 500G |
