# Supplementary material for: Research on digital copyright protection based on the hyperledger fabric blockchain network technology
Source: PeerJ Comput Sci. 2021 Sep 17;7:e709. doi: 10.7717/peerj-cs.709 (PMC8459789; doi:10.7717/peerj-cs.709)
Supplement: Supplemental Information 8 [file peerj-cs-07-709-s008.docx]

| Name | Version |
| --- | --- |
| Linux | Linux version 4.15.0-96-generic 64bit |
| Hyperledger Fabric | Version 1.0 |
