# Supplementary material for: Research on digital copyright protection based on the hyperledger fabric blockchain network technology
Source: PeerJ Comput Sci. 2021 Sep 17;7:e709. doi: 10.7717/peerj-cs.709 (PMC8459789; doi:10.7717/peerj-cs.709)
Supplement: Supplemental Information 9 [file peerj-cs-07-709-s009.docx]

| User name | User ID | User digital copyrights ID | User gender | User Address | User password |
| --- | --- | --- | --- | --- | --- |
| Sean | 522001 | 20191101 | Male | Beijing | 123000 |
| Lexie | 522002 | 2020520、19980722 | Female | Beijing | 112211 |
| Piff | 522003 | 20080101 | Male | Guizhou | 163000 |
