# Supplementary material for: Research on digital copyright protection based on the hyperledger fabric blockchain network technology
Source: PeerJ Comput Sci. 2021 Sep 17;7:e709. doi: 10.7717/peerj-cs.709 (PMC8459789; doi:10.7717/peerj-cs.709)
Supplement: Supplemental Information 10 [file peerj-cs-07-709-s010.docx]

| Digital copyrights name | Digital copyrights ID | Hash value | Owner ID |
| --- | --- | --- | --- |
| Manta | 20191101 | 1819821e05f48422fd98e3600c97d520 | 522001 |
| Hoodie | 2020520 | c9ee101497b7c963186ef5e2259ae6f1 | 522002 |
| Blooming | 19980722 | 3a0f7ad8dd6e3067ae1144722702102a | 522002 |
| tempo | 19980722 | 25bdf0572e88152d53b1a9c2b1ac7dbf | 522003 |
