# Supplementary material for: Research on digital copyright protection based on the hyperledger fabric blockchain network technology
Source: PeerJ Comput Sci. 2021 Sep 17;7:e709. doi: 10.7717/peerj-cs.709 (PMC8459789; doi:10.7717/peerj-cs.709)
Supplement: Supplemental Information 11 [file peerj-cs-07-709-s011.docx]

| Digital copyrights ID | Original owner ID | Current owner ID |
| --- | --- | --- |
| 19980722 | 522001 | 522002 |
