# Supplementary material for: Research on digital copyright protection based on the hyperledger fabric blockchain network technology
Source: PeerJ Comput Sci. 2021 Sep 17;7:e709. doi: 10.7717/peerj-cs.709 (PMC8459789; doi:10.7717/peerj-cs.709)
Supplement: Supplemental Information 12 [file peerj-cs-07-709-s012.docx]

| Scheme | supports the whole life cycle management | Whether a lot of calculation is needed | supports protection of different types of files | Whether the data storage on the link needs to pay fee | supports smart contract |
| --- | --- | --- | --- | --- | --- |
| BMCProtector [27] | $\times$ | √ | $\times$ | √ | $\surd$ |
| Literature [35] | $\times$ | √ | $\times$ | $\times$ | $\times$ |
| DotBlockchain [39] | $\times$ | $\times$ | $\times$ | $\times$ | $\times$ |
| Literature [41] | $\times$ | √ | $\times$ | $\times$ | $\times$ |
| This scheme | √ | $\times$ | √ | $\times$ | $\surd$ |
