# Supplementary material for: Research on digital copyright protection based on the hyperledger fabric blockchain network technology
Source: PeerJ Comput Sci. 2021 Sep 17;7:e709. doi: 10.7717/peerj-cs.709 (PMC8459789; doi:10.7717/peerj-cs.709)
Supplement: Supplemental Information 13 [file peerj-cs-07-709-s013.pdf]

fabric@fabric-pc:~/go/src/github.com/hyperledger/fabric/aberic$ docker ps

CONTAINER ID IMAG COMMAND CREATED STATUS PORTS NAMES

1f762b0036f0 hyperledger/fabric-tools "/bin/bash" 4 minutes ago Up 4 minutes cli

428e9c8de9e5 hyperledger/fabric-peer "peer node start" 5 minutes ago Up 4 minutes 0.0.0.0:7051-7053->7051-7053/tcp peer0.org1.example.com

5d0b820e34d9 hyperledger/fabric-couchdb "tini--/docker-ent…" 5 minutes ago Up 5 minutes 4369/tcp,9100/tcp,0.0.0.0:5984->5984/tcp couchdb

592744861f52 hyperledger/fabric-orderer "orderer" 5 minutes ago Up 5 minutes 0.0.0.0:7050->7050/tcp orderer.example.com
