# Supplementary material for: Research on digital copyright protection based on the hyperledger fabric blockchain network technology
Source: PeerJ Comput Sci. 2021 Sep 17;7:e709. doi: 10.7717/peerj-cs.709 (PMC8459789; doi:10.7717/peerj-cs.709)
Supplement: Supplemental Information 14 [file peerj-cs-07-709-s014.pdf]

2020-05-19 10:21:04.276 UTC [chaincodeCmd] chaincodeInvokeOrQuery -> INFO 077 Chaincode invoke successful. Result: status:200

2020-05-19 10:21:04.276 UTC [main] main ->INFO 078 Exiting……
