# Supplementary material for: Research on digital copyright protection based on the hyperledger fabric blockchain network technology
Source: PeerJ Comput Sci. 2021 Sep 17;7:e709. doi: 10.7717/peerj-cs.709 (PMC8459789; doi:10.7717/peerj-cs.709)
Supplement: Supplemental Information 17 [file peerj-cs-07-709-s017.pdf]

2020-05-19 11:01:39.263 UTC [chaincodeCmd] chaincodeInvokeOrQuery -> INFO 077 Chaincode invoke successful. Result: status:200
2020-05-19 11:01:39.263 UTC [main] main ->INFO 078 Exiting······
