# Supplementary material for: Research on digital copyright protection based on the hyperledger fabric blockchain network technology
Source: PeerJ Comput Sci. 2021 Sep 17;7:e709. doi: 10.7717/peerj-cs.709 (PMC8459789; doi:10.7717/peerj-cs.709)
Supplement: Supplemental Information 22 [file peerj-cs-07-709-s022.pdf]

Error:Error endorsing invoke: rpc error: code=Unknown desc = chaincode error (status: 500, message: asset owner not match) - <nil>
