# Supplementary material for: Research on digital copyright protection based on the hyperledger fabric blockchain network technology
Source: PeerJ Comput Sci. 2021 Sep 17;7:e709. doi: 10.7717/peerj-cs.709 (PMC8459789; doi:10.7717/peerj-cs.709)
Supplement: Supplemental Information 25 [file peerj-cs-07-709-s025.pdf]

Query Result: { “assets” : [“2020520” ,” 19980722”], “id” : “522002” , “name” : “Lexie” }

2020-05-19 12:59:30.042 UTC [main] main -> INFO 008 Exiting……
