# Supplementary material for: Research on digital copyright protection based on the hyperledger fabric blockchain network technology
Source: PeerJ Comput Sci. 2021 Sep 17;7:e709. doi: 10.7717/peerj-cs.709 (PMC8459789; doi:10.7717/peerj-cs.709)
Supplement: Supplemental Information 26 [file peerj-cs-07-709-s026.pdf]

Query Result: { “id” : [“19980722” ,” metadata” : “1271319” ,” name” : “Blooming” }

2020-05-19 13:04:53.464 UTC [main] main -> INFO 008 Exiting……
