# Supplementary material for: Research on digital copyright protection based on the hyperledger fabric blockchain network technology
Source: PeerJ Comput Sci. 2021 Sep 17;7:e709. doi: 10.7717/peerj-cs.709 (PMC8459789; doi:10.7717/peerj-cs.709)
Supplement: Supplemental Information 27 [file peerj-cs-07-709-s027.pdf]

Query Result: [{ “asset_id” : “19980722” ,” origin_owner_id” :” 522001” ,” current_owner_id” :” 522002” }, { “asset_id” : ” 19980722” ,” origin_owner_id” :” originOwnerPlaceholder” , ” current_owner_id” :” 522001” }]
